# Enhancing Community Engagement for Dementia Research in Africa: The Integral role of Community Advisory Boards in Recruitment and Retention

**DOI:** 10.1002/alz.089311

**Published:** 2025-01-09

**Authors:** Temitope Hannah Farombi, Oyedunni Arulogun, Michelle Nichols, Olorunyomi F Olorunsogbon, Timilehin Kumolu, Mayowa Ogunronbi, Taofeek Adedayo Sanni, Victoria Mutiso, Muhammed Uthman, Abdullateef Sule, Emmanuel Assey, Innocent Mwombeki, Joy Onoria, Godspower Chibuke Onunka, Benedict Tagoe, Selem K Melkamu, Ifeoma Adigwe, Adedoyin O Ogunyemi, Solomon Gyabaah, Goldie S. Byrd, Adesola Ogunniyi, Margaret A. Pericak‐Vance, Rufus O. Akinyemi, Africa Dementia Consortium

**Affiliations:** ^1^ University College Hospital, Ibadan Nigeria; ^2^ University of Ibadan, Ibadan, Oyo Nigeria; ^3^ University of South Carolina, South Caroline, SC USA; ^4^ Institute for Advanced Medical Research and Training, College of Medicine, University of Ibadan, Ibadan, Oyo State Nigeria; ^5^ Afe Babalola University, Ado Ekiti, Ekiti Nigeria; ^6^ Africa Mental Health Research and Training Foundation, Nairobi Kenya; ^7^ University of Ilorin, Ilorin, Kwara Nigeria; ^8^ Ahmadu Bello University, Zaria, Kaduna Nigeria; ^9^ Kilimanjaro Christian Tanzania medical Center, Moshi, Moshi Tanzania, United Republic of; ^10^ Kilimanjaro Christian Tanzania Medical center, Moshi, Moshi Tanzania, United Republic of; ^11^ Louise Makerere University, Uganda, Uganda Uganda; ^12^ University of Ghana, Accra, Accra Ghana; ^13^ Addis Ababa University College of Health Sciences, Addis Ababa, Addis Ababa Ethiopia; ^14^ Nnamdi Azikwe University, Nsuka, Enugu Nigeria; ^15^ University of Lagos, Lagos Nigeria; ^16^ Komfo Anokye Teaching Hospital, Kumasi, Kumasi Ghana; ^17^ Maya Angelou Center for Health Equity, Wake Forest University School of Medicine, Winston‐Salem, NC USA; ^18^ University of Ibadan, Ibadan Nigeria; ^19^ John P. Hussman Institute for Human Genomics, University of Miami Miller School of Medicine, Miami, FL USA; ^20^ Neuroscience and Aging Research Unit, Institute of Advanced Medical Research and Training, College of Medicine, University of Ibadan, Ibadan Nigeria

## Abstract

**Background:**

Historically, efforts to engage under‐represented communities in health research have encountered limited success, attributable to inadequate community participation, acceptability, and ownership. Globally, an innovative strategy to foster community involvement in research is the establishment of Community Advisory Boards (CABs). These boards consist of stakeholders from the target community, providing partnership and support throughout all phases of the research, from conception to implementation and evaluation. In pursuit of effective recruitment and retention into the Recruitment and Retention of Alzheimer’s Disease Diversity Genetic Cohorts‐ Alzheimer’s disease Sequencing Project (READD – ADSP) Study, each participating site within the African Dementia Consortium (AfDC) constituted a Community Advisory Board.

**Method:**

A comprehensive standard operating procedure outlining the group’s activities and delineating the composition and role of the CAB within the READD_ADSP project was developed. Eligible members received letters of invitation, and those who agreed were convened for an inaugural meeting to discuss project aims, objectives, and other pertinent details using the SOP as template.

**Result:**

About 14 CAB with 120 members have been established across the nine African sites. Each board comprised a diverse mix of geriatricians, public health leaders, community and religious leaders, retiree organizations, dementia caregivers, NGO dedicated to person living with dementia, media organizations, legal experts, and communication experts. Inaugural meetings at each site demonstrated a high level of interest and motivation to collaborate with the research team for the promotion of recruitment, retention, and advocacy. Using culturally appropriate names for dementia, case identification and referral system, lack of awareness and skilled personnel were among many concerns raised at the cab meetings. There is ongoing to continue to address these concerned raised for effective recruitment at the community.

**Conclusion:**

The strategic partnership with community stakeholders, facilitated through the Community Advisory Boards, proved effective in achieving recruitment and retention goals for dementia research.

